# A prognostic framework for predicting lung signet ring cell carcinoma via a machine learning based cox proportional hazard model

**DOI:** 10.1007/s00432-024-05886-0

**Published:** 2024-07-25

**Authors:** Haixin Chen, Yanyan Xu, Haowen Lin, Shibiao Wan, Lianxiang Luo

**Affiliations:** 1 The First Clinical College, Guangdong Medical University, Zhanjiang, Guangdong 524023 China; 2 Department of Genetics, Cell Biology and Anatomy, University of Nebraska Medical Center, Omaha, NE USA; 3 The Marine Biomedical Research Institute of Guangdong Zhanjiang, School of Ocean and Tropical Medicine, Guangdong Medical University, Zhanjiang, Guangdong 524023 China

**Keywords:** Lung signet ring cell carcinoma, SEER, Nomogram, Machine learning, Cox proportional hazard model

## Abstract

**Purpose:**

Signet ring cell carcinoma (SRCC) is a rare type of lung cancer. The conventional survival nomogram used to predict lung cancer performs poorly for SRCC. Therefore, a novel nomogram specifically for studying SRCC is highly required.

**Methods:**

Baseline characteristics of lung signet ring cell carcinoma were obtained from the Surveillance, Epidemiology, and End Results (SEER) database. Univariate and multivariate Cox regression and random forest analysis were performed on the training group data, respectively. Subsequently, we compared results from these two types of analyses. A nomogram model was developed to predict 1-year, 3-year, and 5-year overall survival (OS) for patients, and receiver operating characteristic (ROC) curves and calibration curves were used to assess the prediction accuracy. Decision curve analysis (DCA) was used to assess the clinical applicability of the proposed model. For treatment modalities, Kaplan-Meier curves were adopted to analyze condition-specific effects.

**Results:**

We obtained 731 patients diagnosed with lung signet ring cell carcinoma (LSRCC) in the SEER database and randomized the patients into a training group (551) and a validation group (220) with a ratio of 7:3. Eight factors including age, primary site, T, N, and M.Stage, surgery, chemotherapy, and radiation were included in the nomogram analysis. Results suggested that treatment methods (like surgery, chemotherapy, and radiation) and T-Stage factors had significant prognostic effects. The results of ROC curves, calibration curves, and DCA in the training and validation groups demonstrated that the nomogram we constructed could precisely predict survival and prognosis in LSRCC patients. Through deep verification, we found the constructed model had a high C-index, indicating that the model had a strong predictive power. Further, we found that all surgical interventions had good effects on OS and cancer-specific survival (CSS). The survival curves showed a relatively favorable prognosis for T0 patients overall, regardless of the treatment modality.

**Conclusions:**

Our nomogram is demonstrated to be clinically beneficial for the prognosis of LSRCC patients. The surgical intervention was successful regardless of the tumor stage, and the Cox proportional hazard (CPH) model had better performance than the machine learning model in terms of effectiveness.

**Supplementary Information:**

The online version contains supplementary material available at 10.1007/s00432-024-05886-0.

## Introduction

Lung cancer remains the leading cause of cancer death (Sung et al. 2021). Common types of lung cancer include adenocarcinoma, squamous cell carcinoma, and small cell lung cancer. Lung adenocarcinoma typically has mutations in the EGFR, KRAS, and ALK genes. LSRCC is a rare adenocarcinoma subtype with a poor prognosis compared with adenocarcinoma. The high percentage expression of TTF-1 and immunostaining profiles CK7+/CK20- in LSRCC was significant in identifying the source of SRCC. Squamous cell carcinoma commonly has mutations in the TP53 and FGFR1 genes. Small-cell lung cancer usually exhibits mutations in the TP53 and RB1 genes (Boland et al. 2014; Campbell et al. 2016; Niu et al. 2022; Chen et al. 2020a, b). According to a 2021 study, lung signet ring cell carcinoma (LSRCC) exhibits distinct risk factors compared to other lung cancers, with a stronger association with Helicobacter pylori and Epstein-Barr virus infections alongside smoking (Boland et al. 2014). Additionally, data from the SEER database indicates that LSRCC accounts for approximately 1–2% of all lung cancer cases (https://seer.cancer.gov/statfacts/html/lungb.html). Furthermore, LSRCC is characterized by the presence of an ALK gene rearrangement and frequent mutations in the ERBB2, TP53, and KRAS genes, contributing to its aggressive behavior and poor prognosis (Akira Okimura et al. 2023) (https://www.cancer.gov/types/lung). LSRCC may more frequently exhibit downregulation of E-cadherin compared to other types of lung cancer. E-cadherin is an important cell adhesion molecule, and its reduction is associated with increased invasiveness and migratory capability of cancer cells Moon et al (2006); Ma et al. (2017). Signet-ring cell carcinoma (SRCC) is a unique subtype of mucin-producing adenocarcinoma characterized by abundant intracellular mucin accumulation, with a poor prognosis across various organs such as the stomach and colon (Wu et al. 2018; Hayashi et al. 1999; Frost et al. 1995; Kitamura et al. 1985; Randolph et al. 1997; Yamashina 1986). While LSRCC can occur as a metastasis from other primary tumors, recent studies have shown a significant rise in primary LSRCC cases, impacting patient survival (Testori et al. 2021); Livieratos et al. 2013). However, more and more studies in recent years have found that the incidence of LSRCC has increased significantly and greatly affects the survival rate of patients. It is believed that early diagnosis and treatment are crucial, with the potential for improved outcomes. LSRCC is a rare non-small cell lung cancer. It is characterized by strong invasion, poor prognosis (Anwar et al. 2020; Hao et al. 2015; Iwasaki et al. 2008), and mostly at an advanced stage (Testori et al. 2021). Its clinical characteristics and treatment have been unclear so far. Normally, after the initial diagnosis, a thorough examination is then carried out to ensure that the tumor is not a metastatic lesion from different primary tumors (Moran 2006). Meanwhile, the prognosis of patients with LSRCC may vary with different treatment methods. So far, there have not been many relevant studies. The clinical knowledge of LSRCC is mainly limited to individual case reports or small case series. There is no large-scale study on the clinicopathological features of LSRCC or the corresponding prognosis of patients (Cai et al. 2021). Based on the above-mentioned, the general prognosis prediction of lung cancer is inaccurate for this kind of population. Therefore, this study aims to explore the significant prognostic factors and establish a more accurate model to predict the survival of patients with LSRCC.

We extracted a large number of population data from the monitoring, epidemiology, and final results (SEER) database, an authoritative cancer statistical database in the United States. The regression analysis was used to determine the influencing prognosis factors and established a nomogram with high accuracy. Machine learning models can combine a large number of variables of different data types in a single model, thereby maximizing the efficacy of prediction testing. Machine learning technology has been widely used to diagnose various types of tumors (Liu et al. 2021). The most typical and commonly used models of machine learning and conventional statistics for cancer-censored survival data are random survival forest (RSF) and Cox proportional hazard model (CPH), respectively. The RSF is an ensemble machine learning method constructed with numerous independent decision trees, each of which receives a random subset of samples and randomly selects a subset of variables at each split in the tree for prediction. The final prediction results of an RSF model are the average of the prediction of each tree. The CPH model is a well-recognized statistical technique to explore the correlation between survival time and covariates (Qiu et al. 2020).

In survival analysis, many different regression modeling strategies can be applied to predict the risk of future events. However, the default choice of analysis often relies on Cox regression modeling due to its convenience. Extensions of the random forest approach (Breiman2001) to survival analysis provide an alternative way to build a risk prediction model (Mogensen et al. 2012). Therefore, the random forest algorithm in machine learning is also used to screen out the most optimized models. Furthermore, we deeply verified the differentiation of nomogram using the pec package in RStudio, while performing cross-validation using bootstrap resampling.

## Materials and methods

### Study population

**Fig. 1:**
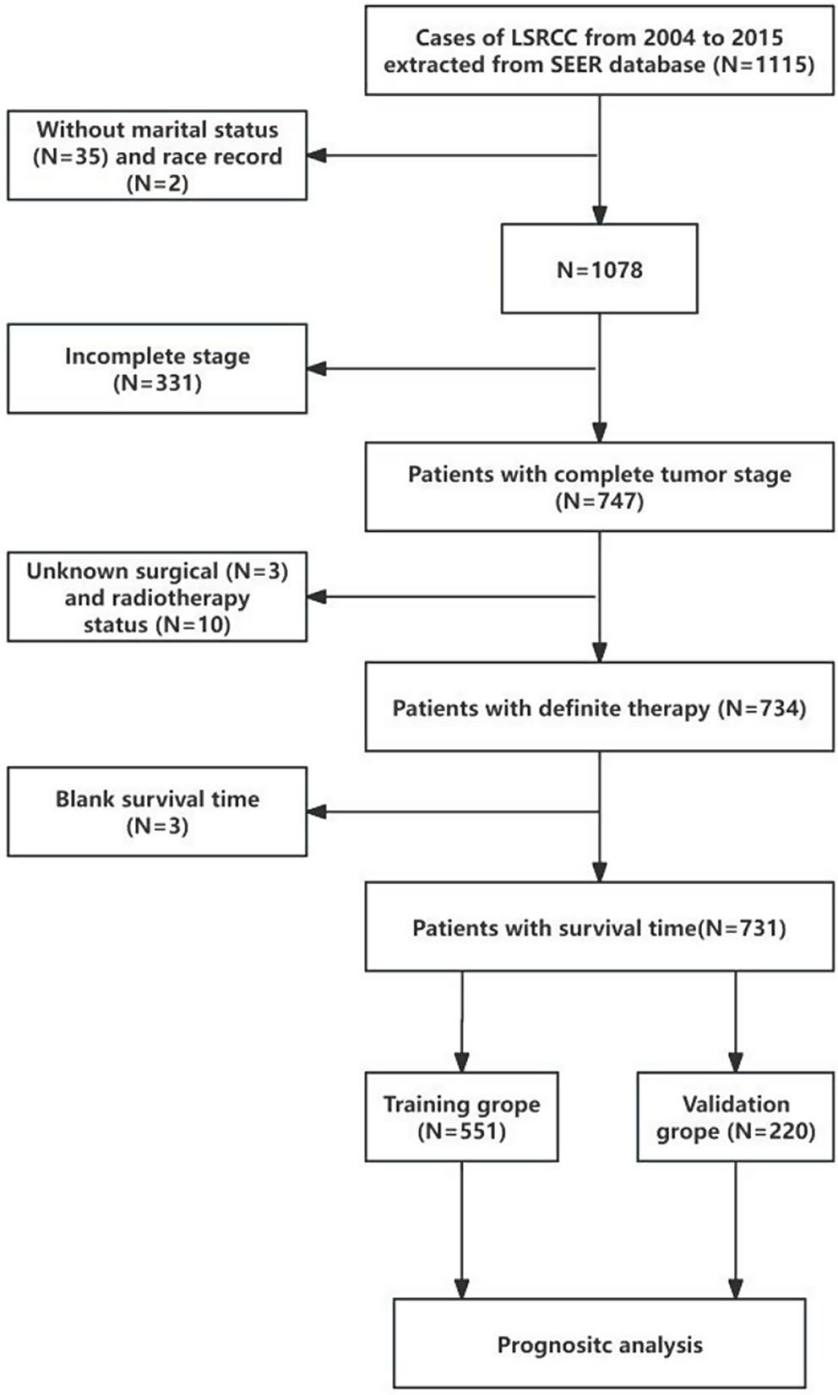
Flowchart of the process of data selection

From Incidence-SEER Research Plus Data 18 Registries, Nov 2020 Sub (2000–2018) in the SEER database, we extracted clinical information of patients diagnosed with LSRCC. All information was obtained from the SEER*Stat program (v 8.3.9). The lesion location was the lung and bronchus, with the year of inclusion as 2004–2015 and SRCC ICD-O-3 encoded as 8490. The extracted variables included patient ID, age, race record, sex, marital status, ICD-O-3 Hist/behave, laterality, primary site, ICD-O-3, T, N and M.Stage, surgery, radiation recode, chemotherapy recode, survival months, vital status recode, SEER cause-specific death classification. Inclusion criteria: The follow-up time for all data was traceable and available. Exclusion criteria: unknown race, unknown marital status, blank T, N, and M.Stage, unknown surgical status, unknown radiotherapy status, unknown survival time, and incomplete data. The inclusion and exclusion process is shown in Fig. 1.

### Age stratification

We extracted a large age range, and to more reliably analyze prognostic and appropriate treatments across ages, we used X-tile software to calculate the age cutoff to transform variable age from continuous variable to categorical variable, dividing patients into three age groups of 22–64 (47.33%), 65–77 (37.76%), 78–85 + (14.91%) (Supplementary Fig. 1).

### Factor exploration and model establishment

Overall survival (OS) and cancer-specific survival (CSS) were predicted using the Kaplan-Meier method and compared by using the log-rank test. OS was defined as the interval from the date of initial diagnosis to the date of death from any cause, and CSS as the survival time from diagnosis to death from lung signet ring cell carcinoma. We randomly divided the data into the training and validation groups at 7:3. The training group was used to build the model and the validation group was used to verify the model. For the training group, we used the Cox proportional hazard regression model and the random forest algorithm to estimate the correlation between clinicopathological features and OS, calculating the risk ratio (HR) and the corresponding 95% confidence interval (Cl).

Significant factors from multivariate Cox regression analysis with factors associated with clinical significance were combined to construct the nomogram, predicting OS in LSRCC patients for 1-year, 3-year, and 5-year.

### Model calibration and discrimination

To investigate how much the grasp of the constructed model has predicted patients’ survival, we evaluated the model with the training group and introduced the ROC curve. The corresponding area under the curve is the degree of the grasp of the model prediction, and the larger the area, the greater the degree of grasp (Park et al. 2004).

Subsequently, we also explained the proximity of the predicted results to the actual situation of the model using the training group calibration graph. Calibration plots were assessed by a calibration curve showing the relationship between predicted probability and observed probability, and the standard curve is a line passing through the coordinate axis origin with a slope of one. The closer the predicted calibration curve is to the standard curve, the better the predictive power of the nomogram (Coutant et al. 2009). In addition, we applied the pec package in RStudio to verify the discrimination of the model, while performing deep cross-validation using resampling. The package provides functions for inverse probability censoring weighted (IPCW) estimation of the time-dependent Brier score and has an option for selecting between ordinary cross-validation, leave-one-out bootstrap, and the 632 + bootstrap for estimating risk prediction performance. It is also possible to compute prediction error curves with independent test data (Mogensen et al. 2012).

### Clinical benefit analysis of the model

Clinical utility is a method to evaluate patients’ prognostic benefits, assessing the extent of benefit using a training group decision analysis curve (DCA). To this end, we introduce “threshold probability” while constructing the DCA curve, and the farther the distance between the model and extreme curves is, the higher the clinical utility of the model (Zhang et al. 2020).

### Validation of the model

We used the data from the validation group to again construct the calibration curve, ROC, and DCA to verify the nomogram to ensure the accuracy of the study.

## Results

### Patient characteristics

We identified 731 LSRCC patients after screening. In our cohort, the highest incidence rate of LSRCC was in patients between 22 and 64 years old (47.33%), followed by patients with 65–77 years old (37.76%) and patients aged 78–85 + years old (14.91%). In general, the majority of patients were young. Among the patients, 53.49% of patients were male, and the rest of them were female (46.51%). There were slightly more male patients than female, but overall, there was no significant difference between them as a whole. There were 82.08% of patients of white race, 10.53% were black people and other ethnic groups accounted for 7.39%. The marital status, more than half of the patients (58.82%) were married. Totally 56.63% of the patients had tumors on the right, 36.94% had on the left and 6.43% had in other positions. Most patients had tumors in the upper lobe (43.78%), followed by the lower lobe (22.98%), NOS (21.61%), middle lobe (6.98%), and main bronchus (4.65%). Most patients (52.26%) were diagnosed with T4, followed by T2 (19.43%), T1 (12.59%), TX (9.58%), and T0 (1.23%). The majority of patients (42.82%) were diagnosed with N2, followed by N0 (21.61%), N3 (21.07%), N1 (8.07%), and NX (6.43%). More than half of the patients had distant metastasis (56.77%) at diagnosis, followed by no distant metastasis (39.67%) and unknown distant metastasis (3.56%). Moreover, surgery was performed in 19.70% of patients, 55.54% of them received chemotherapy, and 35.02% received radiation.

In conclusion, the majority of patients were male, white, and married. The most common LSRCC classifications were tumors on the right laterality and T4, N2, and M1.Stage. In addition, 19.70% of patients received surgery, 55.54% received chemotherapy, 35.02% received radiation, and 24.49% received chemotherapy plus radiation (Table 1).

**Table 1:** Characteristics of LSRCC patients

Characteristic	Overall
*N*	731
**Age (%)**	
22–64	346 (47.33)
65–77	276 (37.76)
78–85+	109 (14.91)
**Sex (%)**	
Female	340 (46.51)
Male	391 (53.49)
**Race (%)**	
Black	77 (10.53)
Other	54(7.39)
White	600 (82.08)
**Marital status (%)**	
Married	430 (58.82)
Single	301 (41.18)
**Laterality (%)**	
Left	270 (36.94)
Other	47 (6.43)
Right	414 (56.63)
**Primary Site (%)**	
Lower lobe	168 (22.98)
Main bronchus	34 (4.65)
Middle lobe	51 (6.98)
NOS	158 (21.61)
Upper lobe	320 (43.78)
**T.Stage (%)**	
T0	9 (1.23)
T1	92 (12.59)
T2	142 (19.43)
T3	36 (4.92)
T4	382 (52.26)
TX	70 (9.58)
**N.Stage (%)**	
N0	158 (21.61)
N1	59 (8.07)
N2	313 (42.82)
N3	154 (21.07)
NX	47 (6.43)
**M.Stage (%)**	
M0	290 (39.67)
M1	415 (56.77)
MX	26 (3.56)
**Surgery (%)**	
No/Unknown	587 (80.30)
Yes	144 (19.70)
**CT (%)**	
No/Unknown	325 (44.46)
Yes	406 (55.54)
**RT (%)**	
No/Unknown	475 (64.98)
Yes	256 (35.02)
**CT + RT (%)**	
No/Unknown	552 (75.51)
Yes	179 (24.49)

*LSRCC* Lung signet ring cell cancer, *CT* Chemotherapy, *RT* Radiotherapy

### Risk factors of OS and CSS

A total of 731 patients were included in the study, and they were randomly assigned to two different cohorts according to the ratio of the training cohort (*n* = 511) and the validation cohort (*n* = 220). To identify the prognostic factors, we performed univariate and multivariate Cox regression analyses in the training cohort.

According to the univariate Cox analysis, we found age, chemotherapy, marital status, primary site, surgery, T.Stage, M.Stage, and N.Stage were significantly associated with OS. Single patients (HR:1.22; Cl: 1.01–1.46) aged 65–77 (HR:1.36, Cl: 1.12–1.67) and 78–85+ (HR:2.01, Cl: 1.54–2.62) years old, diagnosed with advanced T.Stage, having regional lymph node invasion and distant metastasis were associated with a poorer prognosis. The results also demonstrated that compared with patients with primary tumor located in the main bronchus, those in the upper lobe (HR: 0.58, Cl: 0.38–0.89), lower lobe (HR: 0.49; Cl: 0.31–0.76), middle lobe (HR: 0.47; Cl: 0.28–0.80) were all associated with a better prognosis. Patients who underwent surgery (HR: 0.29; Cl: 0.23–0.38) and chemotherapy (HR: 0.64; Cl: 0.53–0.76) had a better prognosis.

Moreover, age, chemotherapy, laterality, primary site, surgery, M.Stage, and N.Stage were independent prognostic factors for CSS. Patients aged 78–85+ (HR:1.80; Cl: 1.36–2.40) years old, having tumors in other positions (HR:1.62, Cl:1.11–2.38), regional lymph node invasion and distant metastasis were associated with a poorer prognosis. Compared with tumors in the main bronchus, upper lobe (HR: 0.29; Cl: 0.19–0.46), lower lobe (HR: 0.30; CI: 0.18–0.48), middle lobe (HR: 0.25; Cl: 0.14–0.44), NOS (HR: 0.43; Cl: 0.27–0.69) were all associated with a better prognosis. Patients who underwent surgery (HR: 0.20; Cl: 0.15–0.27) and chemotherapy (HR:0.73; Cl:0.60–0.89) had a better prognosis (Table 2).

Further, we performed multivariate analysis using Cox proportional hazards regression modeling and found that age, chemotherapy, primary site, surgery, T.Stage, and M.Stage were independent prognostic factors for OS. Patients aged 65–77 (HR:1.56; Cl:1.25–1.94) and 78–85+ (HR:1.80; Cl:1.35–2.39) years old, diagnosed with advanced T.Stage, having distant metastasis were associated with a poorer prognosis. Compared with patients with primary tumor located in the main bronchus, those in the upper lobe (HR: 0.67; Cl: 0.42–1.05), lower lobe (HR:0.55; Cl:0.35–0.88), middle lobe (HR:0.59; Cl:0.34–1.04), NOS (HR:0.60; Cl:0.37–0.98) were all associated with a better prognosis. Patients who underwent surgery (HR: 0.20; Cl: 0.15–0.27) and chemotherapy (HR:0.73; Cl:0.60–0.89) had a better prognosis. According to the results of CSS, eight variables including age, chemotherapy, radiation, primary site, surgery, T.Stage, M.Stage, and N.Stage were identified as independent prognostic. Patients aged 65–77 (HR: 1.24; Cl: 0.99–1.56) and 78–85+ (HR:1.52; Cl:1.11–2.09) years old, diagnosed with advanced T.Stage, having regional lymph node invasion and distant metastasis were associated with a poorer prognosis. Compared with patients with primary tumor located in the main bronchus, those in the upper lobe (HR:0.67; Cl:0.42–1.05), lower lobe (HR:0.44; Cl:0.27–0.71), middle lobe (HR:0.41; Cl:0.23–0.75), NOS (HR:0.40; Cl:0.24–0.67) were all associated with a better prognosis. Patients who underwent surgery (HR:0.25; Cl:0.17–0.36), chemotherapy (HR:0.41; Cl:0.33–0.52), and radiation (HR:0.80; Cl:0.65-1.00) all had a better prognosis (Table 3).

**Table 2:** Univariate analysis of overall survival and cancer special survival

	OS		CSS	
Characteristics	HR (95%Cl)	*P*	HR (95%Cl)	*P*
**Age**				
22–64	Reference		Reference	
65–77	1.36 (1.12–1.67)	0.002	1.00 (0.81–1.23)	0.977
78–85+	2.01 (1.54–2.62)	0.000	1.80 (1.36–2.40)	0.000
**Sex**				
Female	Reference		Reference	
Male	1.15 (0.95–1.37)	0.144	1.09 (0.90–1.33)	0.369
**Race**				
Black	Reference		Reference	
Other	0.67 (0.43–1.05)	0.082	0.65 (0.41–1.03)	0.067
White	0.89 (0.65–1.21)	0.450	0.87 (0.64–1.19)	0.392
**Marital status**				
Married	Reference		Reference	
Single	1.22 (1.01–1.46)	0.036	1.18 (0.97–1.43)	0.103
**Laterality**				
Left	Reference		Reference	
Other	1.35 (0.93–1.96)	0.118	1.62 (1.11–2.38)	0.013
Right	1.16 (0.95–1.41)	0.136	1.08 (0.88–1.33)	0.482
**Primary Site**				
Main bronchus	Reference		Reference	
Upper lobe	0.58 (0.38–0.89)	0.013	0.29 (0.19–0.46)	0.000
Middle lobe	0.47 (0.28–0.80)	0.005	0.25 (0.14–0.44)	0.000
Lower lobe	0.49 (0.31–0.76)	0.002	0.30 (0.18–0.48)	0.000
NOS	0.68 (0.44–1.06)	0.089	0.43 (0.27–0.69)	0.000
**T.Stage**				
T0	Reference		Reference	
T1	0.84 (0.36–1.96)	0.690	0.53 (0.22–1.25)	0.145
T2	1.33 (0.58–3.04)	0.501	0.94 (0.41–2.17)	0.886
T3	1.57 (0.64–3.88)	0.327	1.02 (0.41–2.53)	0.967
T4	2.47 (1.10–5.57)	0.029	2.01 (0.89–4.55)	0.092
TX	2.15 (0.91–5.07)	0.081	2.32 (0.97–5.52)	0.058
**N.Stage**				
N0	Reference		Reference	
N1	1.01 (0.68–1.50)	0.969	1.27 (0.84–1.94)	0.258
N2	1.83 (1.43–2.35)	0.000	2.47 (1.87–3.25)	0.000
N3	1.75 (1.32–2.33)	0.000	2.68 (1.97–3.64)	0.000
NX	2.56 (1.70–3.87)	0.000	3.56 (2.30–5.52)	0.000
**M.Stage**				
M0	Reference		Reference	
M1	1.95 (1.60–2.38)	0.000	2.66 (2.14–3.32)	0.000
MX	2.29 (1.39–3.78)	0.001	2.95 (1.71–5.07)	0.000
**Surgery**				
No	Reference		Reference	
Yes	0.29 (0.23–0.38)	0.000	0.20 (0.15–0.27)	0.000
**Chemotherapy**				
No/Unknown	Reference		Reference	
Yes	0.64 (0.53–0.76)	0.000	0.73 (0.60–0.89)	0.002
**Radiation**				
No/Unknown	Reference		Reference	
Yes	1.00 (0.83–1.21)	0.962	1.04 (0.85–1.27)	0.694

*Cl* Confidence interval, *HR* Hazard ratio, *OS* Overall survival, *CSS* Cancer special survival

**Table 3:** Multivariate analysis of overall survival and cancer special survival

	OS		CSS	
Characteristics	HR(95%Cl)	*P*	HR(95%Cl)	*P*
**Age**				
22–64	Reference		Reference	
65–77	1.56(1.25–1.94)	0.0001	1.24(0.99–1.56)	0.0609
78–85+	1.80(1.35–2.39)	0.0001	1.52(1.11–2.09)	0.0090
**Sex**				
Female	Reference		Reference	
Male	0.95(0.78–1.15)	0.6003	0.98(0.80–1.20)	0.8305
**Race**				
Black	Reference		Reference	
Other	0.81(0.50–1.30)	0.3827	0.69(0.42–1.13)	0.1410
White	1.02(0.72–1.43)	0.9183	0.89(0.64–1.24)	0.4964
**Marital status**				
Married	Reference		Reference	
Single	0.98(0.80–1.20)	0.8292	1.03(0.83–1.28)	0.7689
**Laterality**				
Left	Reference		Reference	
Other	0.82(0.51–1.33)	0.4166	1.11(0.69–1.80)	0.6686
Right	1.02(0.83–1.25)	0.8706	0.92(0.74–1.15)	0.4573
**Primary Site**				
Main bronchus	Reference		Reference	
Upper lobe	0.67(0.42–1.05)	0.0813	0.44(0.27–0.71)	0.0007
Middle lobe	0.59(0.34–1.04)	0.0667	0.41(0.23–0.75)	0.0040
Lower lobe	0.55(0.35–0.88)	0.0130	0.47(0.28–0.77)	0.0030
NOS	0.60(0.37–0.98)	0.0415	0.40(0.24–0.67)	0.0004
**T.Stage**				
T0	Reference		Reference	
T1	1.60(0.62–4.14)	0.3309	1.64(0.61–4.37)	0.3235
T2	1.98(0.78–5.00)	0.1478	2.45(0.96–6.29)	0.0615
T3	2.49(0.92–6.71)	0.0715	2.83(1.02–7.83)	0.0448
T4	3.26(1.33–7.99)	0.0098	3.83(1.53–9.58)	0.0041
TX	2.30(0.90–5.88)	0.0820	3.14(1.18–8.32)	0.0215
**N.Stage**				
N0	Reference		Reference	
N1	1.31(0.85–1.99)	0.2174	1.32(0.85–2.07)	0.2199
N2	1.27(0.95–1.70)	0.1033	1.54(1.11–2.12)	0.0092
N3	1.21(0.86–1.70)	0.2741	1.50(1.03–2.18)	0.0343
NX	1.06(0.65–1.72)	0.8258	1.60(0.97–2.66)	0.0673
**M.Stage**				
M0	Reference		Reference	
M1	1.31(1.04–1.66)	0.0223	1.60(1.25–2.05)	0.0002
MX	1.34(0.75–2.37)	0.3185	1.28(0.69–2.39)	0.4396
**Surgery**				
No	Reference		Reference	
Yes	0.26(0.19–0.38)	0.0000	0.25(0.17–0.36)	0.0000
**Chemotherapy**				
No/Unknown	Reference		Reference	
Yes	0.41(0.33–0.51)	0.0000	0.41(0.33–0.52)	0.0000
**Radiation**				
No/Unknown	Reference		Reference	
Yes	0.83(0.68–1.01)	0.0668	0.80(0.65–1.00)	0.0495

*Cl* Confidence interval, *HR* Hazard ratio, *OS* Overall survival, *CSS* Cancer special survival

### Comparison of the models based on Cox and random forest algorithms

Both in the training and verification set, the C-index based on the Cox model is higher than that of the random forest model, which also reflects that the Cox model has a stronger accuracy in model construction than the random forest algorithm in this study (Table 4).

**Table 4:** C-index-based evaluation of survival prediction with Cox regression and random forest

C-index	Train database	Test database
Cox Regression	0.77(0.74,0.79)	0.75(0.72,0.78)
Random forest survival	0.76(0.68,0.79)	0.74(0.67,0.78)

### Nomogram construction

**Fig. 2:**
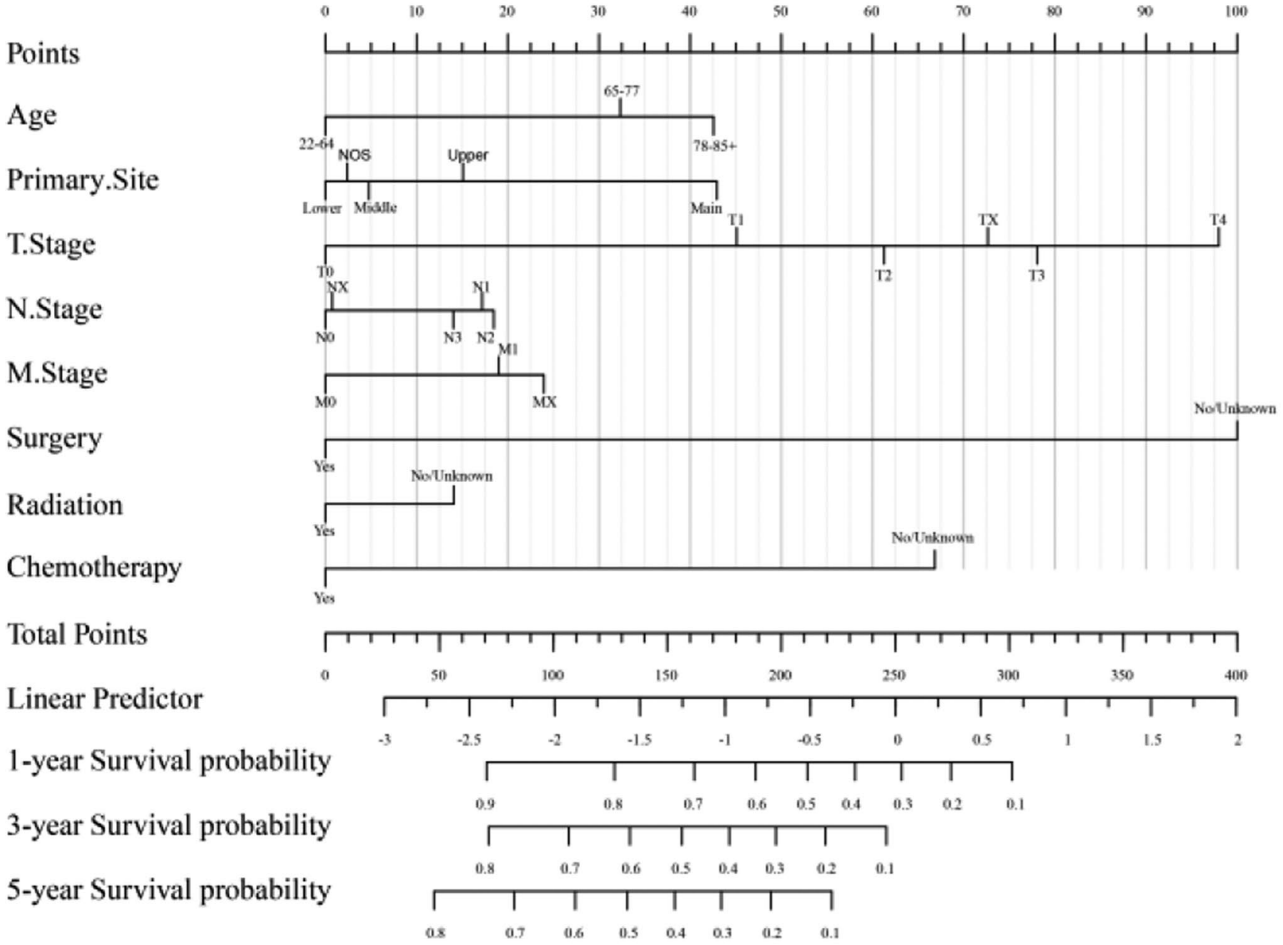
The nomogram of the 1-year, 3-year, and 5-year overall survival of patients in the training cohort

Age, chemotherapy, primary site, surgery, T.Stage, and M.Stage were identified as independent prognostic factors via multivariate Cox analysis (all *p* < 0.05) and further included to establish the nomogram. However, it is important to consider both clinical and statistical significance when choosing inclusion variables (Iasonos et al. 2008). Therefore, we also included radiation and N-Stage in the predictive model (Fig. 2). To use the nomogram, an individual patient’s value is located on each variable axis, and a line is drawn upward to determine the number of points received for each variable value. The sum of these numbers is located on the total points axis, and a line is drawn downward to the survival axis to determine the likelihood of 1-year, 3-year, and 5-year survival time (Zheng et al. 2019). The nomogram revealed that surgery, T.Stage, and chemotherapy had the largest impact on the patient’s prognosis.

### Deep verification

Model 1 was constructed using six significant factors derived from Cox multivariate analysis and two other clinically relevant factors, and six significant factors obtained from Cox multivariate analysis were used to construct model 2. Compared the predictive power of the two models and validated while performing deep cross-validation using bootstrap repeat sampling. The results showed that the C-index of model 1 was slightly higher before and after cross-validation, thus its prediction power was also stronger (Supplementary Fig. 2).

## Survival analysis

### A population analysis of different therapy modalities

**Fig. 3:**
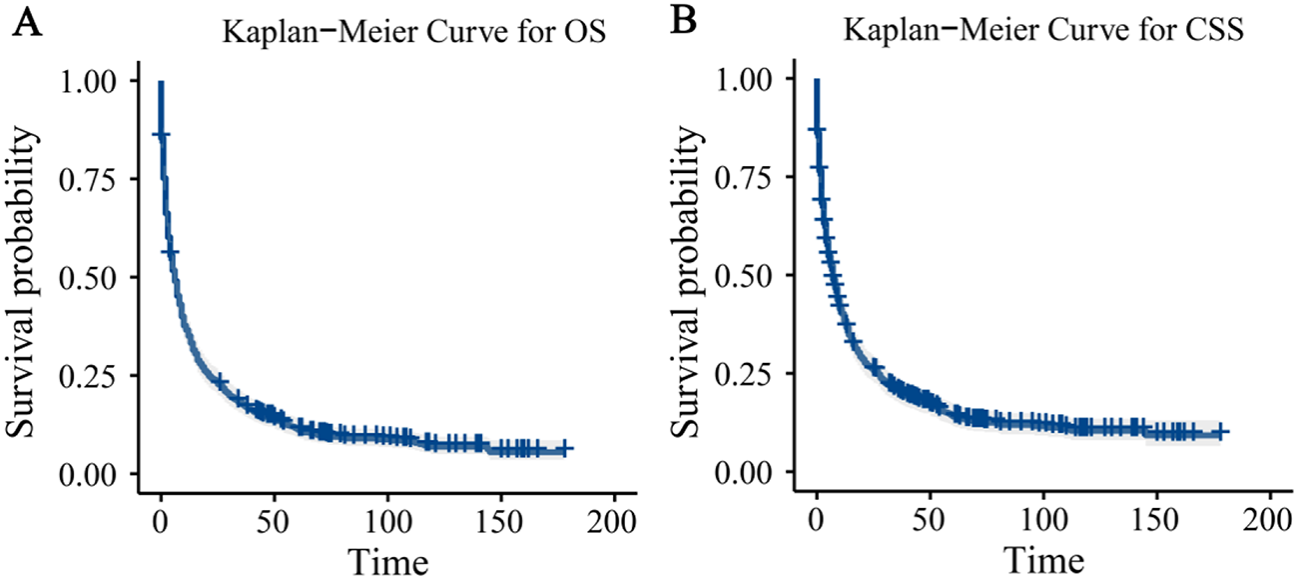
Kaplan-Meier survival curves for all patients. (**A**) Overall survival. (**B**) Cancer-specific survival

Using Kaplan-Meier curves, we compared the OS and CSS in LSRCC patients (Fig. 3A and B), and the results showed that the CSS was similar to the OS.

**Fig. 4:**
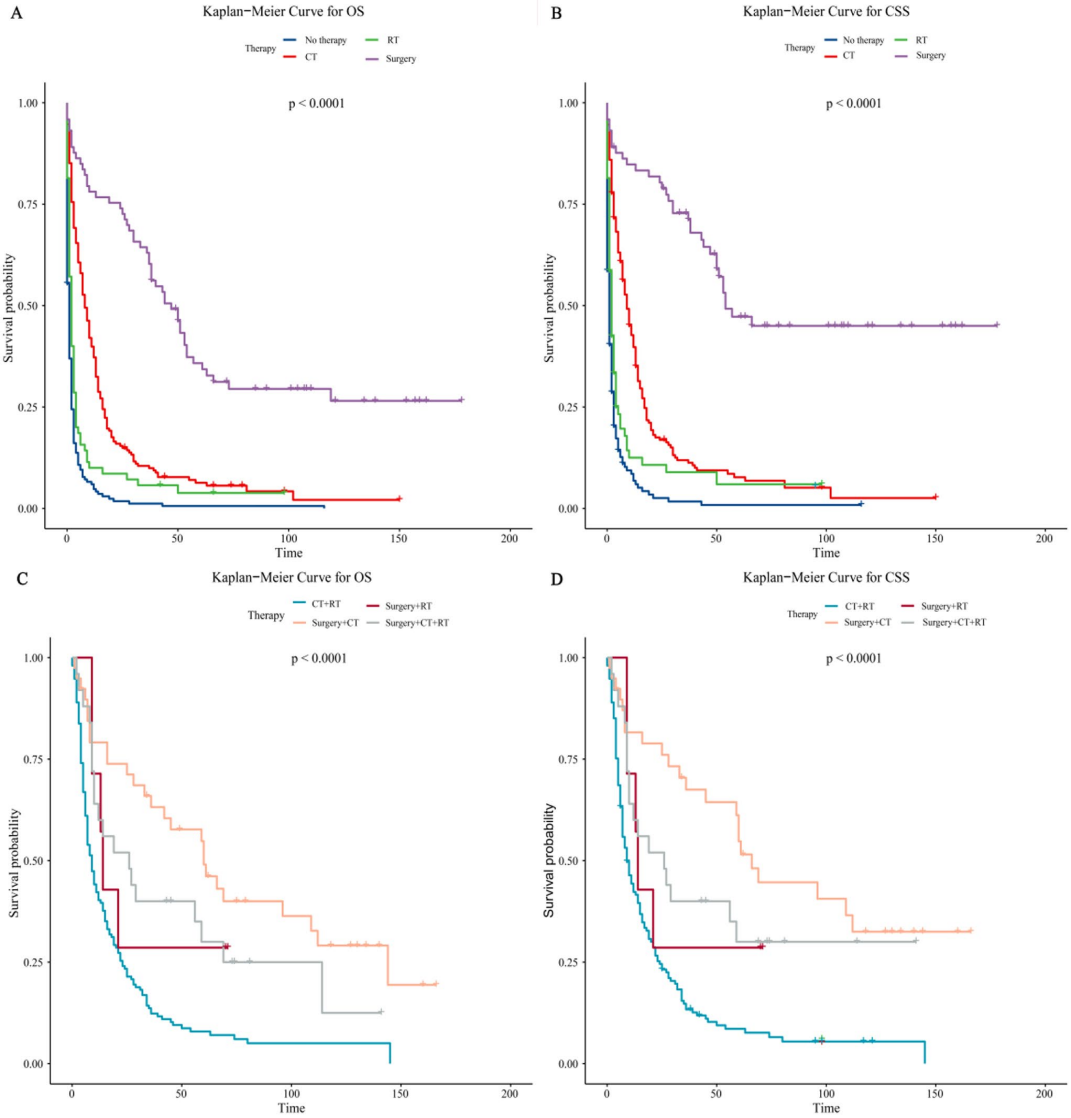
Survival in patients undergoing monotherapy. (**A**) Overall survival. (**B**) Cancer-specific survival. Survival of patients receiving combination therapy. (**C**) Overall survival. (**D**) Cancer-specific survival

In the same way, we used Kaplan-Meier curves to compare the effects of different therapy modalities (including surgery, radiotherapy, and chemotherapy) on patients’ survival (Fig. 4). The results showed that among monotherapy, analyzing the results of OS, we found that patients who received surgery only were associated with the best prognosis, followed by chemotherapy only and radiotherapy only. The patients who received no therapy demonstrated the worst prognosis (Fig. 4A). Also, we found a similar conclusion in the results of CSS (Fig. 4C). Among the combined therapy modalities, no matter whether in OS (Fig. 4B) or CSS (Fig. 4D), surgery combined with chemotherapy had the best prognosis overall, followed by surgery combined with chemoradiotherapy and surgery combined with radiation. Patients who received radiation combined with chemotherapy had the worst prognosis. However, patients who received surgery combined with radiation had the greatest survival benefit for a short time after therapy is worth noting. Combining the Kaplan-Meier curves of OS and CSS for analysis, we found a common conclusion that surgical intervention had better prognoses on OS and CSS in all monotherapy or combined therapy modalities listed.

### Age stratification for different therapy modalities

**Fig. 5:**
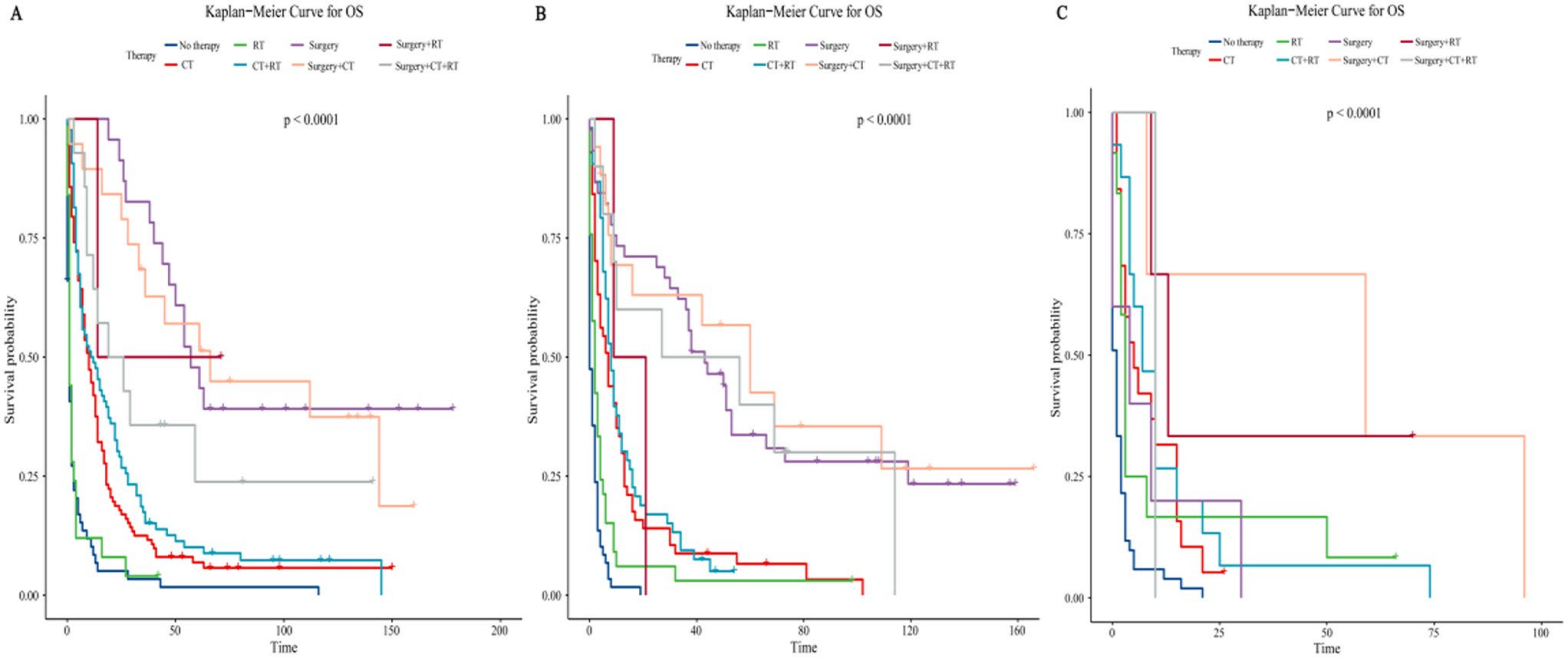
Overall survival of patients in different age groups. (**A**) Aged 22–64. (**B**) Aged 65–77. (**C**) Aged 78–85+

The age data we screened had a large span, considering that therapy for younger patients might not be appropriate for older patients, therefore, we used Kaplan-Meier curves to analyze the therapy of different age groups (Fig. 5). For patients aged 22–64 (Fig. 5A), on the whole, therapy with surgical intervention had a greater benefit on survival. In the long run, the results of surgery alone and surgery combined with chemotherapy are considerable. Surgery combined with radiation yielded similar effects to surgery alone in the short term but is not appropriate for long-term survival effects. The effect of surgery combined with chemoradiotherapy was worse than the other three therapy modalities with surgical intervention. Radiation alone and chemoradiotherapy had a similar effect in the short term after therapy, and subsequently, chemoradiotherapy had a slightly better effect than chemotherapy alone. Patients without therapy and who received radiation only were associated with the worst prognostic. For patients aged 65–77 (Fig. 5B), overall, therapy with surgical intervention is more beneficial for survival, excluding surgery combined with radiation. In the long term, we found that surgery combined with chemotherapy, surgery combined with chemoradiotherapy, and chemotherapy only are considerable. The therapy of surgery combined with radiation contributed to higher short-term survival but did not apply to long-term survival. Among the therapies without surgical participation, the survival effect of chemoradiotherapy was similar to that of chemotherapy alone. Patients with chemotherapy alone had a longer survival period than those who received chemoradiotherapy. Patients who received no therapy and radiation alone were associated with a poorer prognosis, but radiation alone had a longer survival period than no therapy. For patients aged 75–85+ (Fig. 5C), different from the previous two groups, the two modalities of therapy -- surgery combined with radiation and surgery combined with chemotherapy had considerable and long-term results, while the effect of surgery alone and surgery combined with chemoradiotherapy was not ideal. The effects of monotherapy modalities focused on short survival. Patients with no therapy had the worst prognosis.

For LSRCC patients, the aggressive therapeutic intervention was associated with a better prognosis, and the younger patients had a relatively longer survival and greater benefit.

### Different monotherapy modalities of T.Stage

**Fig. 6:**
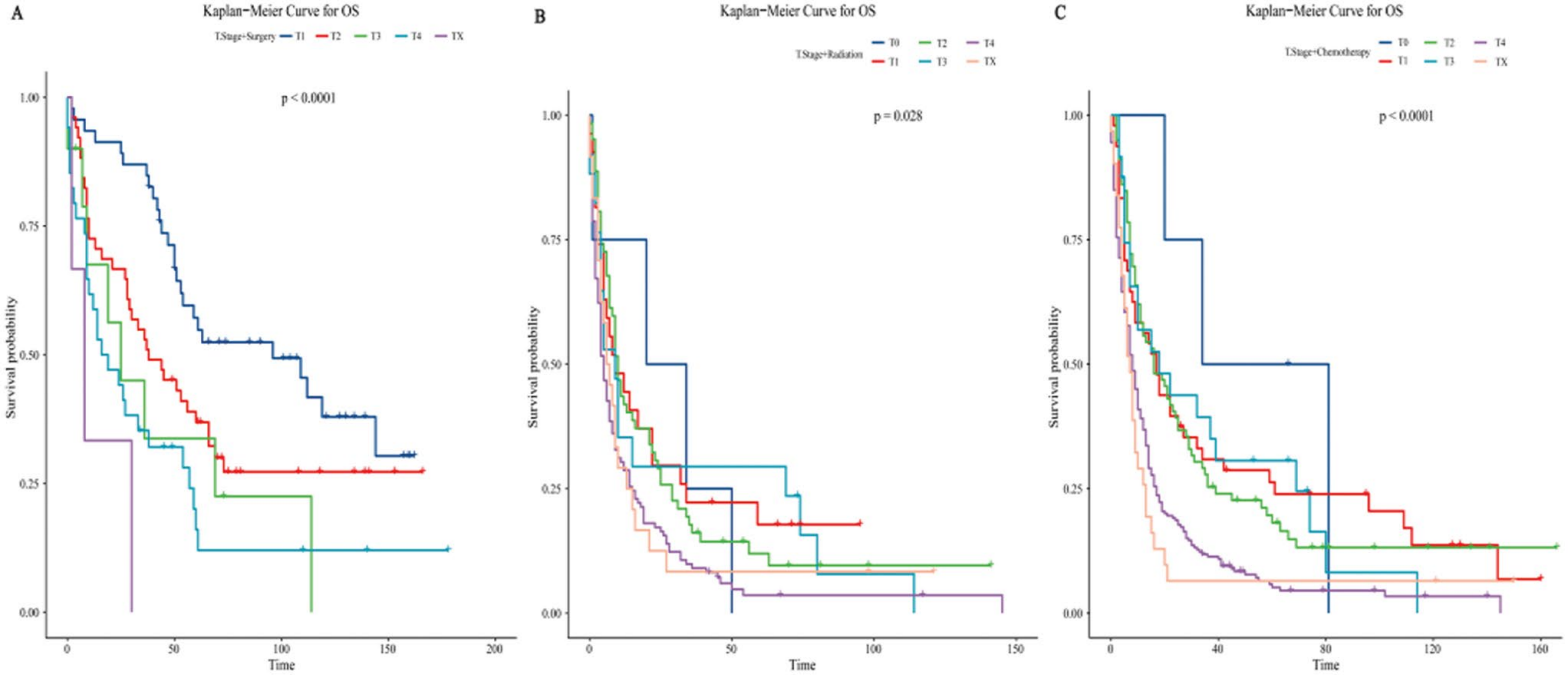
Overall survival of patients with different T.Stage. (**A**) Surgery only. (**B**) Radiotherapy only. (**C**) Chemotherapy only

Clinical T.Stage is commonly used to indicate the tumor size, in general, the larger the T.Stage is, the larger the tumor volume is. Similarly, the tumor size can also have an impact on the therapy mode and consequence to some extent. Therefore, to investigate the effect of tumor size on the outcome of different therapy modalities, we included T.Stage with three monotherapy modalities into Kaplan-Meier curves and found significant differences in prognosis under different treatment modalities (Fig. 6). For patients who received surgery alone (Fig. 6A), we found there was no T0 patient in this therapy modality. Patients with smaller T-Stage had a better prognosis overall, but in some particular cases, T3 had a slightly better prognosis than T2, and T4 also had a better prognosis than T3, but the differences were not significant. As is shown in Fig. 6A, patients diagnosed with TX appear to be associated with shorter survival and the worst prognosis, which may be related to the inability to identify the primary tumor. Subsequently, we analyzed patients who received radiation alone (Fig. 6B), and the analysis showed that the prognostic benefit of T0 was significantly optimized over the other stages during the initial period of survival. Meanwhile, patients diagnosed with T3 had a better prognosis than T0, T1, and T2 during some certain survival time. As a whole, patients diagnosed with T4 had the worst prognosis, confirming the logic that larger tumors are more burdensome for patients. Finally, in the chemotherapy-only group (Fig. 6C), the prognosis of patients diagnosed with T0 was significantly improved compared with other T-Stages. Patients diagnosed with T1 had a greater benefit than T2 overall, but patients diagnosed with T2 and T3 also had a better prognosis than T1 in a certain period. Meanwhile, patients diagnosed with T4 had a better prognosis than that of TX at first in a survival period, and then in the following period, the prognosis benefit of TX was slightly better than that of T4. In summary, patients diagnosed with T0 had a relatively better prognosis overall, regardless of the therapy modality. No matter in what T-Stage, patients who underwent radiation were associated with the worst prognosis.

### Calibration and validation of the nomogram

**Fig. 7:**
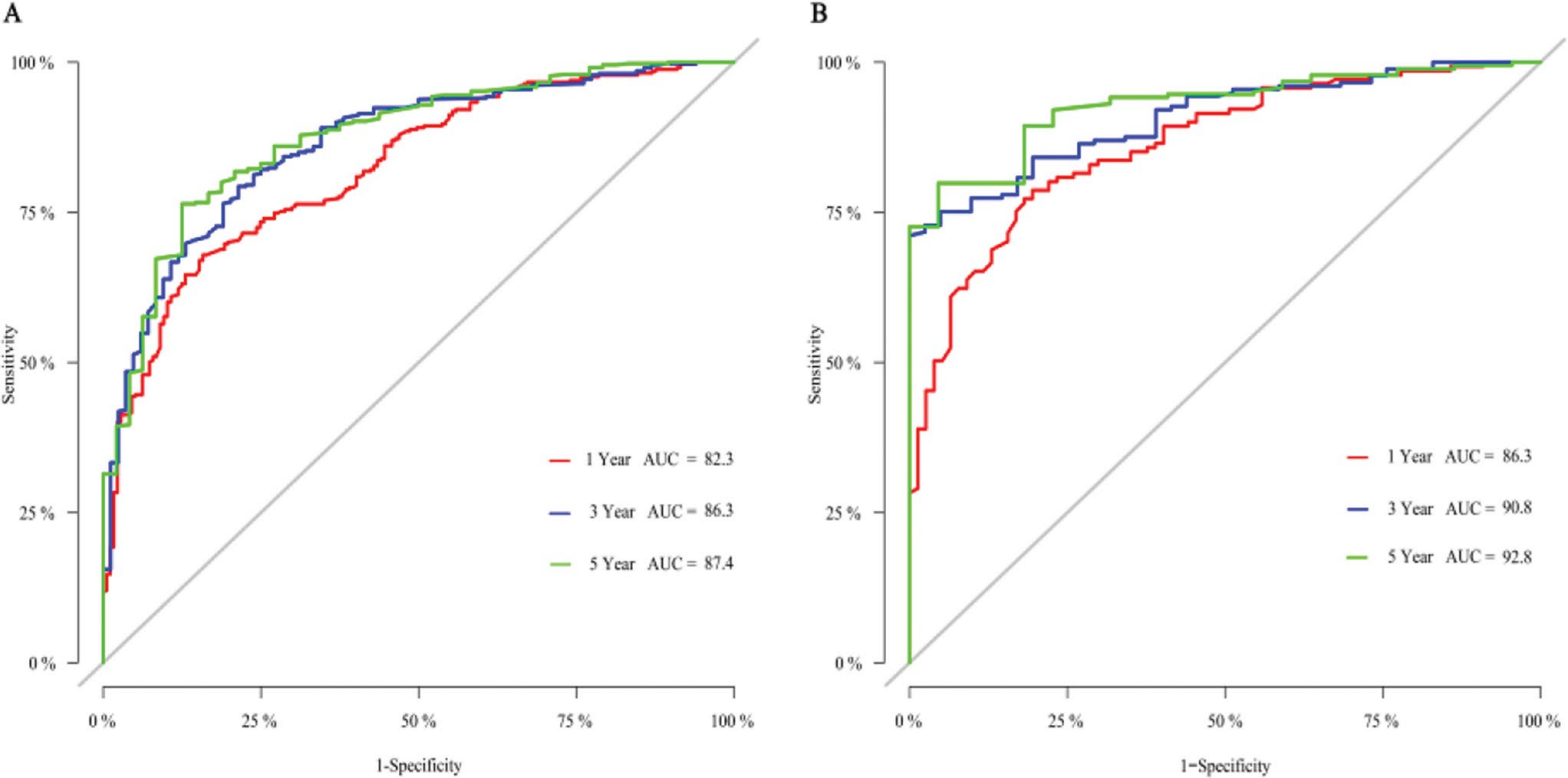
ROC curves and AUC at 1-year, 3-year, and 5-year. (**A**) Training cohort. (**B**) Validation group

**Fig. 8:**
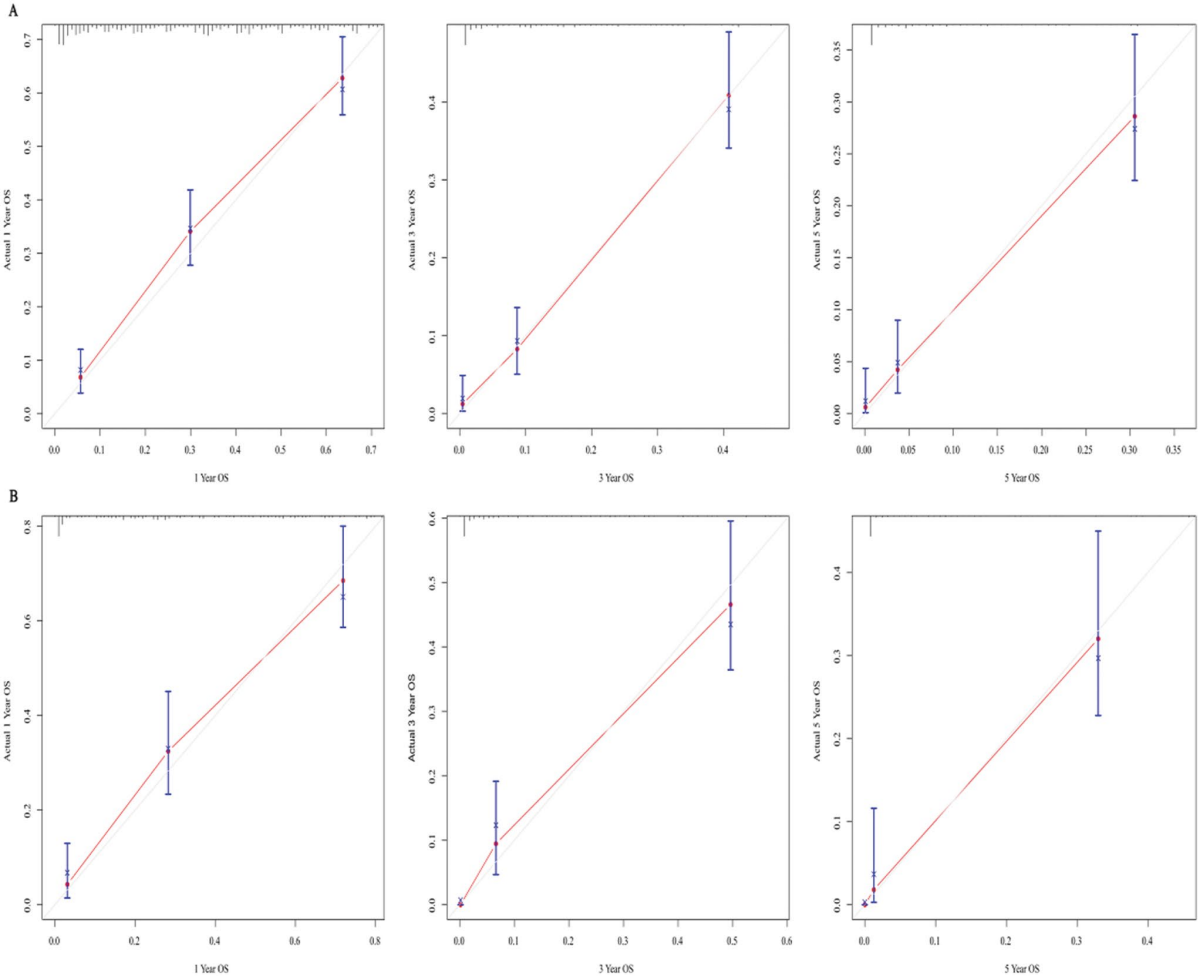
Calibration curves predicting the 1-year, 3-year, and 5-year overall survival of patients. (**A**) Training cohort. (**B**) Validation group

The ROC curve of the training group (Fig. 7A) showed that the AUC value constructed by us was 82.3% (Cl:78.7-85.9%) in 1-year, 86.3% (Cl:82.3-90.3%) in 3-year and 87.4% (Cl:82.7- 92.2%) in 5-year, indicating the considerable predictive power of the model we built. The prediction result of the nomogram was close to the standard curve of the training group, demonstrating that the nomogram prediction result was close to the actual situation (Fig. 8A, B, C).

External validation of the nomogram showed that the AUC value of the validation group was 86.3% (Cl:81.5-91.1%) in 1-year, 90.8% (Cl:86.9-94.7%) in 3-year, 92.8% (Cl:88.8- 96.8%) in 5-year, which further demonstrated that the predictive power of our model was desirable (Fig. 7B). The calibration curve of the validation group further confirmed that the results predicted by the nomogram were close to the actual situation (Fig. 8D, E, F).

### Clinical benefits of the nomogram

**Fig. 9:**
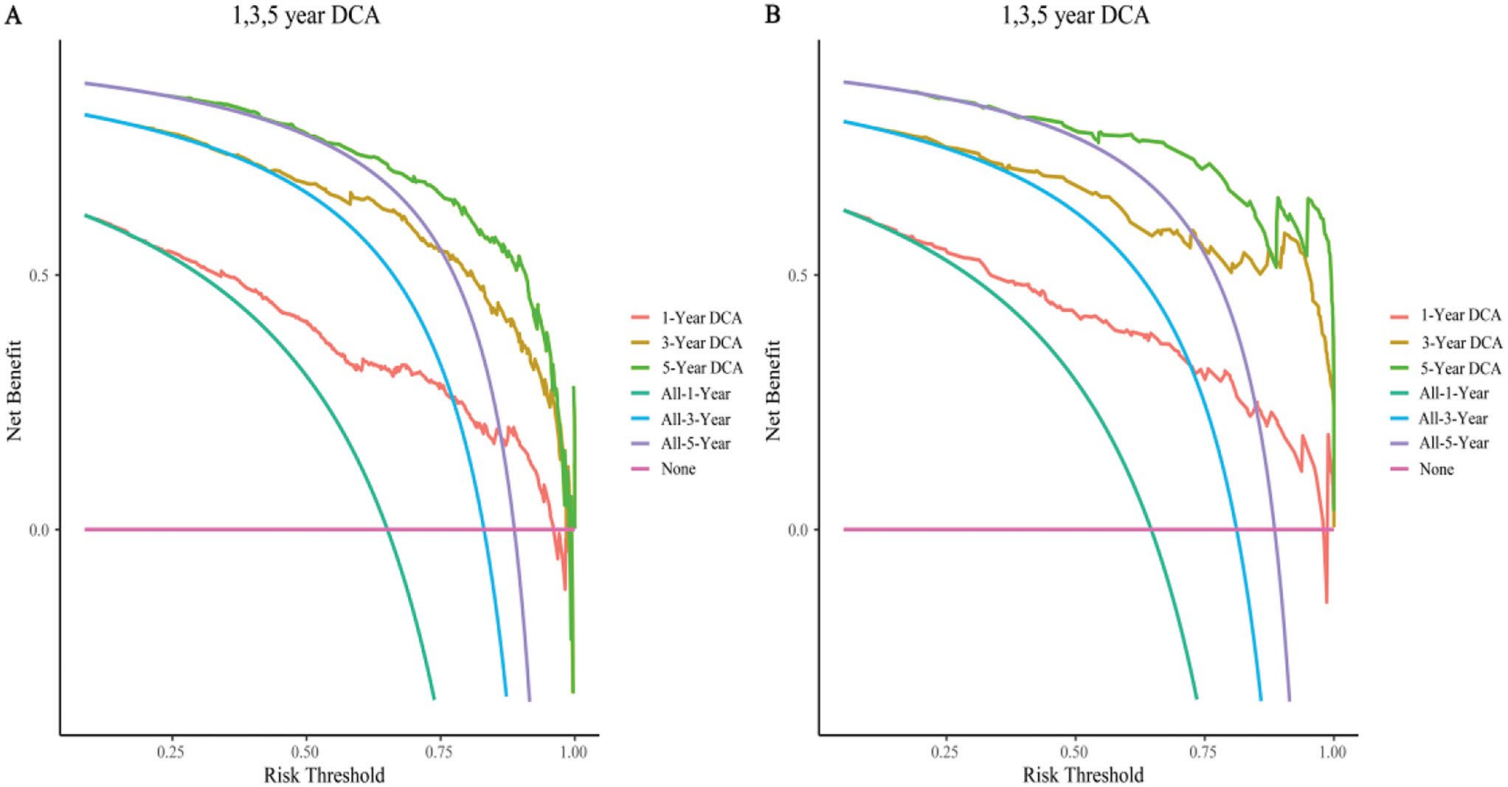
The DCA of the nomogram to 1-year, 3-year, and 5-year overall survival. (**A**) Training cohort. (**B**) Validation cohort

The DCA results for the nomogram are shown in Fig. 9. According to the DCA, when triggering the medical intervention at the same threshold probability, the nomogram brought greater net benefit to patients and excellent clinical utility (Fig. 9A). The DCA curve of the validation group further supported this conclusion (Fig. 9B).

## Discussion

Due to the rarity of LSRCC and its lack of specific clinical manifestations, it is very difficult to diagnose. It is often confused with other types of lung cancer, leading to misdiagnosis, and treatment delays, and conventional treatments are often ineffective (Cai et al. 2021). Recent research offers a glimmer of hope. Owing to its unique biology, existing prognostic tools for lung cancer are unsuitable for LSRCC patients (Anwar et al. 2020). However, studies show promise for targeted therapies and immunotherapies (Boland et al. 2014; lbrahim Yildiz 2021). ALK inhibitors have presented an opportunity for the individualized treatment of lung cancers and provided an alternative therapeutic approach for those patients intolerant to chemotherapy and radiation therapy (Hao et al. 2015; Kwak et al. 2010). A case report shows that Lorlatinib has an antitumor effect in ALK-positive LSRCC (lbrahim Yildiz 2021). Lorlatinib is a potent, brain-penetrating, third-generation, macrocyclic ALK/ROS1 TKI with broad-spectrum potency against most known resistance mutations that develop during treatment with existing first- and second-generation ALK TKIs (lbrahim Yildiz 2021). Depending on the biopsy site and tumor types, the vacuoles of signet ring cells seem to contain quantities of mucin, glycogen, lipid, or immunoglobulin (Yiğit et al. 2018). Historically, research on LSRCC has been limited, with case reports and small analyses dominating the field (Cai et al. 2021). However, a recent rise in SRCC diagnoses across various primary sites has revealed significant variations in survival based on tumor location (Wu et al. 2018). Therefore, further study is urgently needed. A nomogram is a useful and convenient tool for individualized cancer prognoses, which is widely used for cancer prognosis (Iasonos et al. 2008; Liang et al. 2015; Kattan et al. 2002). We wanted to develop a nomogram to explore significant factors influencing LSRCC prognosis as well as more appropriate treatment modalities. On the nomogram, treatment mode accounts for a relatively large proportion and has a greater impact on prognosis. Therefore, our study further analyzed the Kaplan-Meier survival curves of OS and CSS in different treatment modalities. In the age-stratified Kaplan-Meier survival curve analyzing different treatment modalities, overall, surgery combined with chemotherapy was associated with a better prognosis. With aging, fewer treatment approaches to benefit patients, and poorer prognostic outcomes for patients receiving treatment, we speculate that this may be linked to poor basal physical condition in older patients. By analyzing Kaplan-Meier survival curves for age-stratified and T-Stage-stratified patients, we can provide a basis for what treatment methods different patients should receive. Regular physical examination, early tumor detection and actively cooperating with treatment yielded better survival benefits even in older patients, consistent with the results obtained in the previous study (Wang et al. 2020).

Random forests have had incredible success across a variety of learning disciplines and have fared well in machine-learning competitions (Deo 2015). We also constructed the prognostic model using the random forest algorithm and compared it with the Cox risk regression model. We found that the Cox proportional hazard model outperformed the random forest model in terms of predictive accuracy. This finding is consistent with previous research that has shown that the Cox model is a robust and reliable tool for survival prediction (Poon and Lu 2022; Chen et al. 2020a, b; Moolgavkar et al. 2018). The superior performance of the Cox model in this study may be due to several factors. First, the Cox model is less sensitive to outliers and missing data than the random forest model (Wang and Li 2017; Baralou et al. 2023; Wang et al. 2023). Second, the Cox model is able to capture complex nonlinear relationships between the predictor variables and the outcome variable. Future research should focus on validating our findings in larger and more diverse patient populations. Additionally, future research should investigate the use of the Cox model in conjunction with other machine learning techniques to further improve the accuracy of patient prognosis prediction.

For the nomogram we constructed, the pec package in RStudio was applied in it. Based on the random forest algorithm, owing to LSRCC being a rare cancer, it is difficult to get the best effect in the calculation process, and there exists experience of overfitting (Liu and Dai 2022).

Of course, our study also has certain limitations. First, as a regression inquiry, some bias is inevitable. Second, SRRC is a rare type of cancer, and the patient’s clinical characteristics are not complete in the SEER database. So the blank and absence of a large number of data are inevitable. For example, the data of the Grade variable is mostly blank, so it can not be explored. Third, the latest installment of AJCC is version 8, while the TNM installment of LSRCC patients is still blank even in version 7, so we had to study AJCC version 6. Fourth, the SEER database does not have some variable comorbidities, and chemotherapy regimens, which may hinder further prognostic analysis. Finally, other independent large-scale datasets are lacking to externally validate the models.

## Conclusions

For LSRCC patients, we can construct a nomogram to predict their prognosis, and after examination and adjustment, the nomogram has certain predictive power and clinical significance. Further, we analyzed the influence of therapy modalities on the prognosis of patients with different clinical characteristics. The prognosis of patients with early diagnosis and interventional therapy was significantly improved, and surgical intervention had better effects on OS and CSS. Moreover, compared with the two prediction models, the model constructed based on Cox is more reliable than that of the random forests.

## Electronic supplementary material

Below is the link to the electronic supplementary material.

Supplementary Material 1

## Data Availability

All data are in the article.
