# Supplementary material for: A prognostic framework for predicting lung signet ring cell carcinoma via a machine learning based cox proportional hazard model
Source: J Cancer Res Clin Oncol. 2024 Jul 25;150(7):364. doi: 10.1007/s00432-024-05886-0 (PMC11272739; doi:10.1007/s00432-024-05886-0)
Supplement: Supplementary file 1 — Supplementary Material 1 [file 432_2024_5886_MOESM1_ESM.docx]

Supplementary Material

**Construct prognostic analysis and prediction model of lung signet ring cell carcinoma with machine learning and cox proportional hazard model**

**Haixin Chen^1†^, Yanyan Xu^1†^, Haowen Lin^1^, Lianxiang Luo^2,3,4*^**

*** Correspondence:** Lianxiang Luo : luolianxiang321@gdmu.edu.cn

## Supplementary Figures


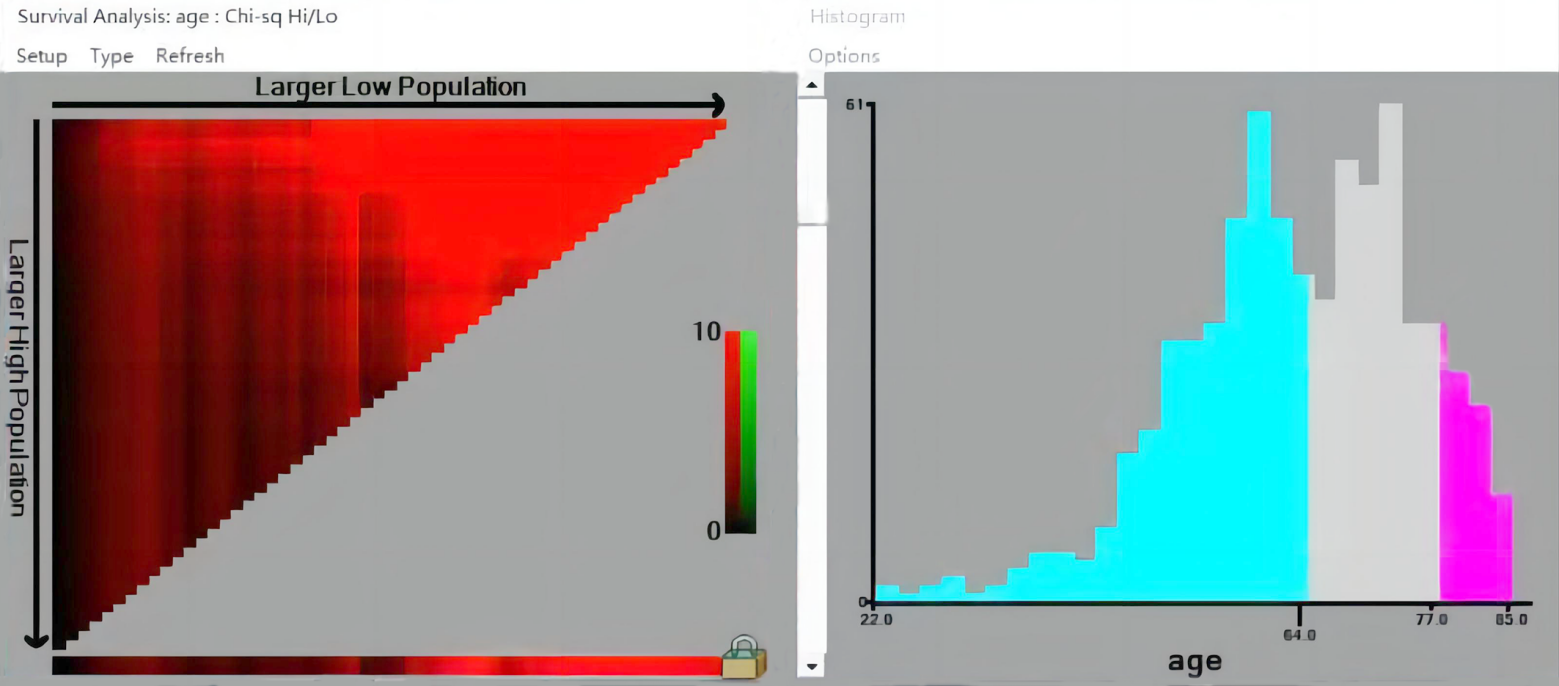


**Supplementary Figure 1.** Identification of optimal cut-off values of age via X-tile software analysis.


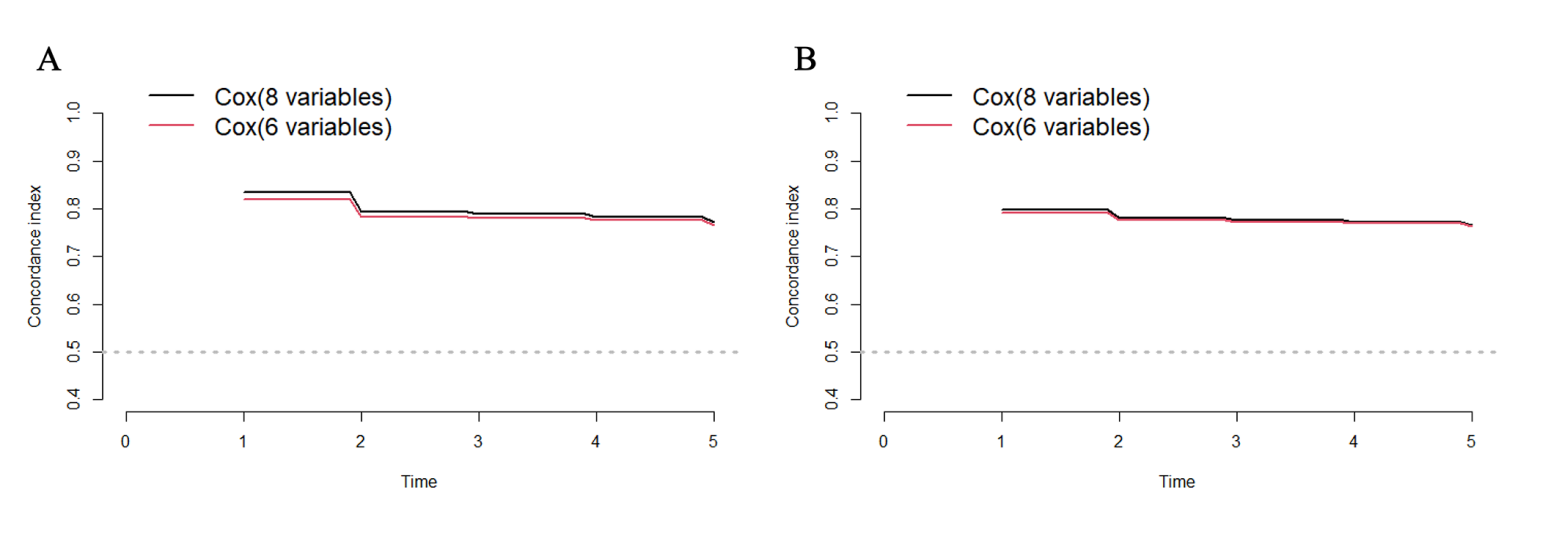


**Supplementary Figure S2.** Comparison with the ability to predict survival of model 1 and model 2. **(A)**Before cross-validation. **(B)** After cross-validation.
